# Myofibroma/myofibromatosis: MDCT and MR imaging findings in 24 patients with radiological-pathological correlation

**DOI:** 10.1186/s12880-020-00498-9

**Published:** 2020-08-26

**Authors:** Kun-ming Yi, Kang Chen, Qiang Ma, Lu Wang, Ran Li, Yi Wang

**Affiliations:** 1 Department of Radiology, Daping Hospital, Army Medical University, Chongqing, 400042 China; 2 Chongqing Clinical Research Centre of Imaging and Nuclear Medicine, Chongqing, 400042 China; 3 Department of Radiology, First Affiliated Hospital, Army Medical University, Chongqing, 400038 China; 4 Department of Pathology, Daping Hospital, Army Medical University, Chongqing, 400042 China

**Keywords:** Myofibroma, Myofibromatosis, Multidetector computed tomography, Magnetic resonance imaging, Pathology

## Abstract

**Background:**

The aim of this study was to characterize the radiological features of myofibroma on multidetector computed tomography (MDCT) and magnetic resonance imaging (MRI) and correlate the imaging findings with pathologic features.

**Methods:**

The radiological findings of 24 patients with 29 myofibromas were retrospectively reviewed. All images were evaluated with emphasis on density, signal intensity, hypointense area, and enhancement, correlating these with pathologic findings.

**Results:**

On plain MDCT scan, 4(26.7%) tumors were homogeneous isodensity, 4(26.7%) tumors were heterogeneous hyperdensity, and 7(46.7%) tumors were heterogeneous hypodensity. On contrast-enhanced MDCT scan, all tumors (9/9) showed heterogeneous enhancement with moderate in 3(33.3%) and marked in 6(66.7%) tumors, and their enhancements were higher compared to adjacent skeletal muscle (*P* = 0.0001). On MRI, heterogeneous slight hyperintensity, homogeneous slight hyperintensity, and heterogeneous hypointensity on T1-weighted imaging (T1WI) were observed in 14(82.3%), 1(5.9%) and 2(11.8%) tumors, respectively. On T2-weighted imaging (T2WI) and fat-suppressed (FS) T2WI, all tumors demonstrated heterogeneous hyperintensity. All tumors showed heterogeneous marked enhancement on FS contrast-enhanced T1WI. On T1WI, T2WI, FS T2WI, and FS contrast-enhanced T1WI, irregular strip or/and patchy hypointensities were found in 16(94.1%), 12(100%), 17(100%) and 17(100%) tumors, respectively, and pseudocapsule was seen in 5(29.4%) tumors. The hypointensities and pseudocapsule on MRI were exactly corresponding to pathological interlacing collagen fibers and fibrosis. The age of the recurrent group was lower than that of the non-recurrent group (*P* = 0.001) and the tumors without pseudocapsule were more likely to recur than those with pseudocapsule (*P* = 0.034).

**Conclusion:**

Myofibromas are characterized by heterogeneous density or signal intensity, with moderate or marked enhancement. The hypointensities and pseudocapsule on MRI may be helpful in diagnosis, and the absence of pseudocapsule and younger age may be risk factors for tumor recurrence.

## Background

Myofibroma/myofibromatosis is an uncommon disorder of fibroblastic/myofibroblastic proliferation with perivascular myoid differentiation and is one of the most common benign fibrous tumors in infancy and childhood [1–3]. Currently, in the WHO classification of tumors of soft tissue and bone, myofibroma/myofibromatosis was classified as part of pericytic/perivascular tumors, the term myofibroma was used to denote solitary lesions and the term myofibromatosis to designate those multicentric [4]. The genetic etiology of the disorder remains uncertain, both autosomal dominant and autosomal recessive inheritance have been reported, the mutation in *PDGFRB*, *NOTCH3* and *PTPRG* are associated with autosomal dominant multicentric myofibromatosis [5, 6].

Clinically, myofibromas frequently present as slowly growing, asymptomatic single mass or diffuse multiple nodules in the dermis and subcutaneous tissues of the head and neck region, although they can also affect the trunk, extremities, skeletal muscles, bone or internal organs [2, 7]. Most patients occur in neonates or infants during the first decade of life, however, they have also been reported in adults [7, 8]. There is a slight male predominance, with a reported male/female ratio of 1.3–2.4:1 [2, 7–9]. The greatest diameter of myofibromas ranged from 0.3 to 17.8 cm [7, 8, 10–12]. Additionally, three clinical types of myofibroma are recognized: (1) solitary type (a single lesion in the skin, subcutaneous tissue, muscle, bone or viscus), (2) multicentric type (multicentric lesions limited to skin, subcutaneous tissues, muscles, and bones, without visceral involvement), (3) generalized type (multicentric lesions with visceral involvement) [2, 13]. In these types of myofibroma, the solitary form is the most common type, multicentric type with nonvisceral lesions accounts for the majority of familial-related myofibromatosis [2]. Spontaneous involution is common, the solitary type and multicentric type with single (or limited) visceral lesion have a better prognosis than the multicentric type with multiple visceral lesions [2, 14].

Radiological examinations can be helpful in assessing the extent of the myofibroma/myofibromatosis and its progression and prognosis. However, the plain radiography, computed tomography and magnetic resonance imaging (MRI) findings of myofibroma are mostly case reports and small series [11, 12, 15–30], and some imaging findings in our study have rarely or never been reported. Our study aimed to describe the multidetector computed tomography (MDCT) and magnetic resonance imaging (MRI) findings of the myofibroma and to correlate them with pathologic features, in order to improve the diagnostic accuracy of preoperative radiological examinations.

## Methods

### Clinical data

Our institutional ethics committee approved this study and waived the need for individual consent due to its retrospective nature. Between January 2011 and December 2018, all patients with myofibroma/myofibromatosis were identified at our institutions. Finally, a series of 24 patients with preoperative radiological examinations were enrolled and their first preoperative radiological images were reviewed. Of these 24 patients, MDCT and MRI scans were obtained in 12 and 15 patients, respectively, and 3 of them underwent both MDCT and MRI scans. Clinical characteristics including age, sex, anatomic locations, and types of the tumor, clinical symptoms and signs, surgical and pathological findings, treatment, and outcomes, were obtained through a review of medical records.

The diagnosis was confirmed by review of pathology slides of all cases. All specimens were fixed in 4% buffered formalin, routinely processed, and then embedded in paraffin; 4-μm-thick sections were prepared and stained with hematoxylin and eosin (HE). Immunostaining was performed in all the patients for smooth muscle actin (SMA), desmin, S-100 protein, CD34, Calponin, and Ki-67.

### MDCT protocols

MDCT was performed in 12 patients by using either of the following medical imaging equipments: the 64–MDCT LightSpeed scanner (GE Healthcare, Milwaukee, WI, USA) and the 16–MDCT Emition scanner (Siemens, Erlangen, Germany). For contrast-enhanced MDCT scans, the non-ionic contrast agent (Ultravist, Bayer Schering Pharma; Omnipaque, GE Healthcare) + 30 mL normal saline was intravenously injected through the antecubital vein at a rate of 2.5–4.5 mL/s. Contrast agent volume was administered at 2 mL/kg body weight, and the upper dose limit was 120 mL for every patient. Of these 12 patients, triple-phase contrast-enhanced MDCT scans were performed for 3 patients with 4 lesions (the locations included the penis in 1 patient with 2 lesions, and the other 2 lesions were located in the psoas and abdominal wall, respectively), images of the arterial, venous and delayed phases were acquired at 24–30, 45–60 and 120 s, respectively, after contrast agent injection. Dual-phase contrast-enhanced MDCT images were obtained for 5 patients with 5 lesions (the lesions were located at the neck in 1 patient and maxillofacial region in 4 patients), with arterial and venous phases at 20–25 and 50–55 s, respectively. Scan parameters were as follows: tube voltage 100–120 kV and tube current 100–320 mA, 3 or 5-mm-thick slice reconstructions, matrix 512 × 512, field of view 120–500 mm. All MDCT studies performed axial and coronal reformations, the sagittal reformations were performed selectively.

Both soft-tissue and bone windows were reviewed. Both unenhanced and enhanced MDCT scans were performed for 8 patients with 9 tumors, only enhanced was performed for 1 patient with 1 tumor, and unenhanced MDCT scans were obtained in 3 patients with 5 tumors. Attenuation at the soft-tissue window was measured on the workstation in the MDCT images before and after contrast administration in the same section, areas of cystic degeneration, calcification and necrosis were avoided. Mean density values were recorded in Hounsfield units (HU).

### MRI protocols

Fifteen patients underwent MRI examination. Among them, 12 patients were performed with a 1.5-T system (Magnetom Aera, Siemens, Erlangen, Germany; Avanto, Siemens, Erlangen, Germany) and 3 patients with a 3.0-T system (Magnetom Verio, Siemens, Erlangen, Germany; Magnetom Trio TIM, Siemens, Erlangen, Germany; Magnetom Spectra, Siemens, Erlangen, Germany). In addition, the section thickness was 3 mm, the intersection gap was 1 mm, the field of view was 120–380 mm. In all patients, spin-echo (SE) T1-weighted imaging (T1WI) (repetition time/echo time [TR/TE], 450–750 ms/9–12 ms), fat-suppressed (FS) fast SE T2-weighted imaging (T2WI) (TR/TE, 3000–9000 ms/35–86 ms), and FS gadolinium-enhanced SE T1WI (TR/TE, 530–750 ms /9–12 ms) were obtained. Gadolinium-enhanced SE T1WI (TR/TE, 450–752 ms /9–10 ms) or T2WI (TR/TE, 3600–4350 ms /36–80 ms) was obtained in some cases. Axial, coronal, or sagittal images were performed with the above sequences. Contrast-enhanced SE T1WI or/and FS contrast-enhanced SE T1WI were obtained after the intravenous injection of a standard dose of 0.1 mmol/kg body weight of gadopentetate dimeglumine (Magnevist; Bayer Healthcare). Both plain radiography and MRI scans were obtained in 4 patients.

### Image viewing and evaluation

Two experienced radiologists (K.Y., Y.W., with 9 and 24 years of experience in diagnostic imaging of the musculoskeletal system, respectively) performed the imaging analysis independently on picture archiving and communication system (PACS). In cases of discrepancies in the interpretations of the two radiologists, a consensus was reached. The radiological findings were evaluated for the following characteristics: (1) number (solitary or multifocal); (2) locations; (3) shape (round, oval, or lobular); (4) margins (well-defined or poorly defined); (5) size (maximal diameter); (6) density, signal intensity and enhancement patterns on MDCT/MRI scans (homogeneous or heterogenous, compared with the adjacent skeletal muscle); (7) hypointense area on MRI scans (defined as irregular strip or/and patchy hypointensities within the tumor on all MRI sequences); (8) pseudocapsule (defined as a complete or incomplete low signal rim at the periphery of the lesion on T1WI, T2WI, and FS T2WI); (9) the degrees of enhancement on MDCT scans included three types according to the increase in CT values: mild (10 to < 30 HU), moderate (30–50 HU), and marked (> 50 HU), and tumor enhancements were also compared with that of the adjacent skeletal muscle; (10) calcifications, fat, haemorrhage, necrosis, and cysts.

### Statistical analysis

We stratified the patients with follow-up data into the recurrence group and non-recurrent group. Statistical analyses were performed with software (SPSS, version 20; SPSS, Chicago, IL, United States). An independent sample *t*-test was used to compare the differences between the enhancement values of tumors and adjacent skeletal muscles on MDCT and assess the difference between the age of the recurrence group and the non-recurrent group. The Mann-Whitney test was used to compare the differences in the maximum diameter of the tumors between the two groups. The difference of tumor recurrence between tumors with and without pseudocapsule was compared using Fisher exact test. The difference A *P* value of less than 0.05 was considered to indicate a significant difference.

## Results

### Clinical findings

The clinical characteristics are summarized in Table 1. In total, 24 patients (mean age, 32.3 ± 16.5 years; range, 8–64 years) with 29 tumors were identified in our study, including 7 men and 17 women. Of all patients, extremities (15, 51.7%) were the most common sites, followed by the head (6, 20.7%), trunk (6, 20.7%), and neck (2, 6.9%). Twenty-one patients (87.5%) presented solitary lesion and 3 patients (12.5%) showed multicentric involvement. In 2 patients, each of them had 3 lesions, one patient showed the lesions located in the bilateral hips and the right thigh (case 5) and another patient located in right thigh (case 21). Two subcutaneous tumors were found at the penis (case 11) in one patient. The main symptoms of the tumor mainly included painless swelling, enlarging mass, nodule, local pain, and limp.

**Table 1:** Clinical characteristics of 24 patients with myofibroma

Case	Location	Duration of symptoms before admission (M)	Type of myofibroma	Treatment	Follow-up
1	Hip	12	Solitary	Excision+ radiotherapy+ chemotherapy	Recurrence
2	Foot	6	Solitary	Excision	Recurrence
3	Hip	7	Solitary	Excision	Recurrence
4	Calf	2	Solitary	Excision	No recurrence
5	Right hip Right thigh Left hip	180	Multifocal	Excision Excision Excision	No recurrence No recurrence No recurrence
6	Upper arm	36	Solitary	Excision	Recurrence
7	Neck	36	Solitary	Biopsy	Lost to follow-up
8	Foot	9	Solitary	Excision	No recurrence
9	Maxillary sinus	5	Solitary	Excision	No recurrence
10	Calf	48	Solitary	Excision	Recurrence
11	Penis Penis	6	Multifocal	Excision Excision	No recurrence No recurrence
12	Upper arm	68	Solitary	Excision	Recurrence
13	Rectus abdominis	3	Solitary	Excision	No recurrence
14	Pre-auricular	3	Solitary	Excision	No recurrence
15	Thigh	20	Solitary	Excision	Recurrence
16	Psoas	6	Solitary	Biopsy	Lost to follow-up
17	Iliac fossa	2	Solitary	Partial excision	Lost to follow-up
18	Maxillofacial	10	Solitary	Excision	No recurrence
19	Mandible	0.5	Solitary	Excision	No recurrence
20	Neck	1	Solitary	Excision	No recurrence
21	Right thigh Right thigh Right thigh	72	Multifocal	Excision Excision Excision	No recurrence No recurrence No recurrence
22	Pre-auricular	12	Solitary	Excision	No recurrence
23	Nasal vestibule	6	Solitary	Excision	No recurrence
24	Lumbar back	24	Solitary	Excision	No recurrence

### MDCT findings

**Fig. 1:**
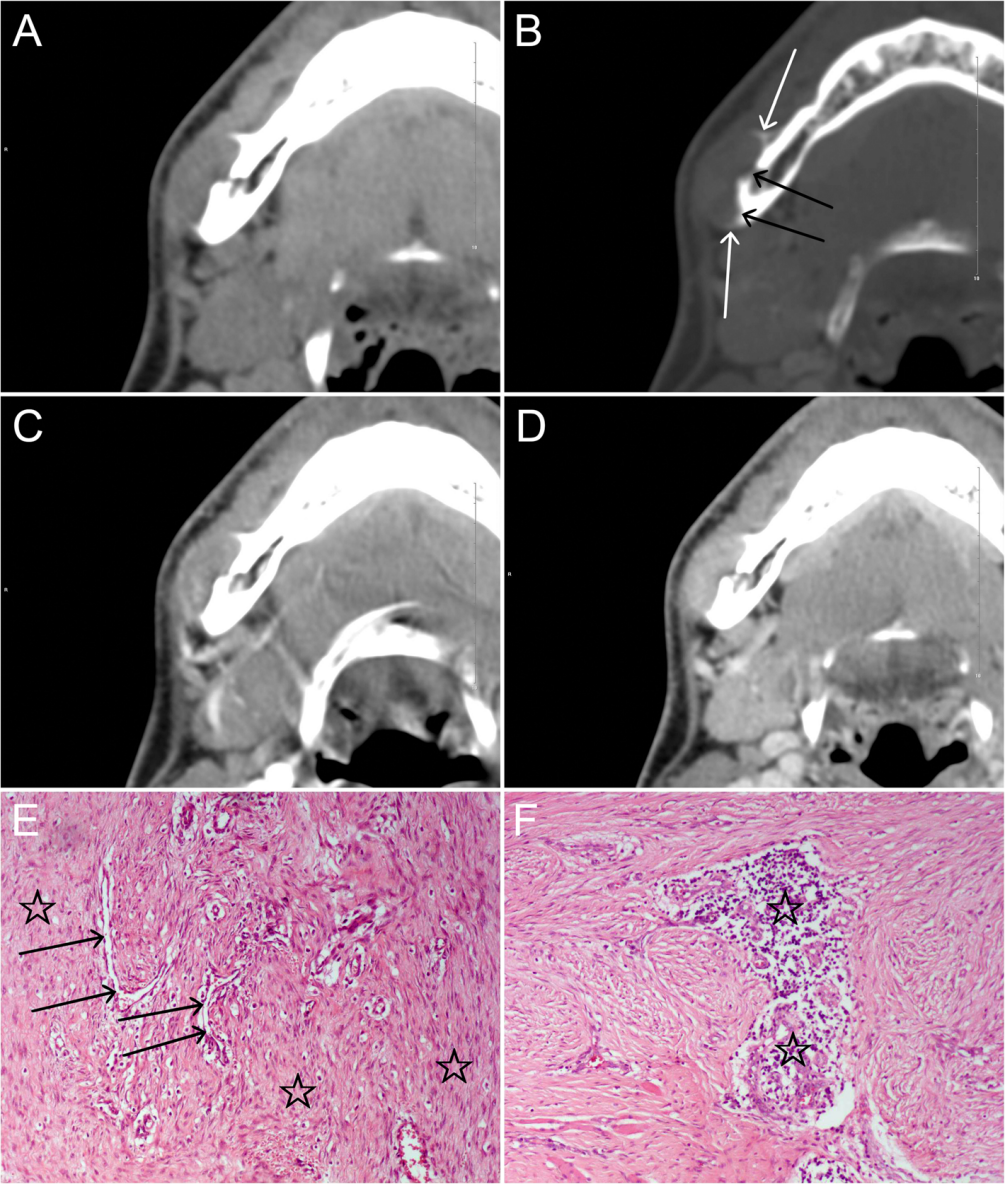
A young patient in his twenties with a myofibroma in the right body of the mandible. **a** and **b**: Axial unenhanced MDCT images show a well-defined round lesion on the lateral margin of the mandible, with periosteal reaction (white arrows) and resorption of the external cortical bone (black arrows). **c** and **d**: The lesion shows marked enhancement on axial arterial- and venous-phase contrast-enhanced MDCT images and greater enhancement is seen in the venous phase. **e**: Photomicrograph (HE, × 100) demonstrates a large number of interlacing bundles of spindle-shaped cells (☆) with tapered blunt-ended nuclei and eosinophilic cytoplasm; hemangiopericytoma-like pattern of the blood vessels (arrows) is focally seen. **f**: Inflammatory cells (☆) are also present in the lesion (HE, × 100)

The MDCT features of 15 myofibromas in 12 patients are summarized in Table 2. In the 15 tumors, 4 (26.7%) were round or round-like masses with well demarcated, the remaining 11 (73.3%) tumors were lobulated masses with ill-defined margins. Their maximal diameter ranged from 0.9 to 28.5 cm (median 5.3, interquartile range [IQR] 2.4–14.4). In all tumors, 3(20%) involved both subcutaneous and adjacent tissues, including skeletal muscle (2, 13.3%) and parotid gland (1, 6.7%). One (6.7%) out of the 15 tumors occurred in left maxillary sinus with adjacent bone erosion, others involved subcutaneous tissue (4, 26.7%), skeletal muscle (5, 33.3%), parapharyngeal space (1, 6.7%), and mandible (1, 6.7%) (Fig. 1), respectively. Of the 15 tumors evaluated by MDCT, 4(26.7%) were homogeneous isodensity relative to the adjacent skeletal muscle, 4(26.7%) showed heterogeneous hyperdensity and 7(46.7%) appeared as heterogeneous hypodensity on the unenhanced MDCT images. 3(20%) out of the 15 tumors showed multiple irregular nodular calcifications at the bilateral buttocks and the right thigh in the same patient (case 5), and 5(33.3%) exhibited necrosis within these tumors.

**Table 2:** Radiological findings in the 24 patients with myofibroma

Radiological findings	No. (%) of lesions/median (IQR)	
	MDCT (*n* = 15)	MRI (*n* = 17)
Locations		
Head	4 (26.7%)	2 (11.8%)
Neck	1 (6.7%)	1 (5.9%)
Extremities	6 (40%)	12 (70.6%)
Trunk	4 (26.7%)	2 (11.8%)
Density (*n* = 15)		
Isodensity; homogeneous	4 (26.7%)	
Iso-and high- density; heterogeneous	4 (26.7%)	
Iso-and low- density; heterogeneous	7 (46.7%)	
Necrosis	5 (33.3%)	
Calcifications	3 (20%)	
Enhancement on MDCT (*n* = 9)		
Mild	0	
Moderate	3 (33.3%)	
Marked	6 (66.7%)	
T1WI (*n* = 17)		
Homogeneous hypointensity		1 (5.9%)
Heterogeneous hyperintensity		14 (82.3%)
Heterogeneous hypointensity		2 (11.8%)
T2WI (*n* = 12)		
Homogeneous hyperintensity		0
Heterogeneous hyperintensity		12 (100%)
FS T2WI (*n* = 17)		
Homogeneous hyperintensity		0
Heterogeneous hyperintensity		17 (100%)
FS enhanced T1WI (*n* = 17)		
Homogeneous enhancement		0
Heterogeneous enhancement		17 (100%)
Size		
Maximal diameter [cm; median (IQR)]*	5.3 (2.4–14.4)	4.5 (2.9–12.7)

*Unless otherwise noted, data are presented as number (%). MDCT, multidetector computed tomography; MRI, magnetic resonance imaging; T1WI, T1-weighted imaging; T2WI, T2-weighted imaging; FS, fat-suppressed; IQR, interquartile range

Attenuation data could be measured for 14 tumors with unenhanced scans and 9 of them with enhanced scans. In our series, the mean attenuation was 42.2 ± 10.8 HU (*n* = 14) on non-enhanced images, 96.8 ± 42.4 HU (*n* = 9) on the arterial phase images, 111.2 ± 39.5 HU (*n* = 9) on the venous phase images, and 122 ± 44.8 HU (*n* = 4) on the delayed phase images. The mean maximum enhancement value of the tumors was 78.9 ± 34.8 HU, and all actual enhancement were significantly higher than that of the adjacent skeletal muscle (11.7 ± 3.1 HU) (*P* = 0.0001). Tumor enhancements were moderate in 3(33.3%) and marked in 6(66.7%) (Fig. 1). All these 9 tumors (9/9) showed heterogeneous enhancement, 7(77.8%) of them presented progressive enhancement, and 2(22.2%) showed decreased enhancement in the latest phase (i.e. venous or delayed) of the enhanced scans.

### MRI findings

**Fig. 2:**
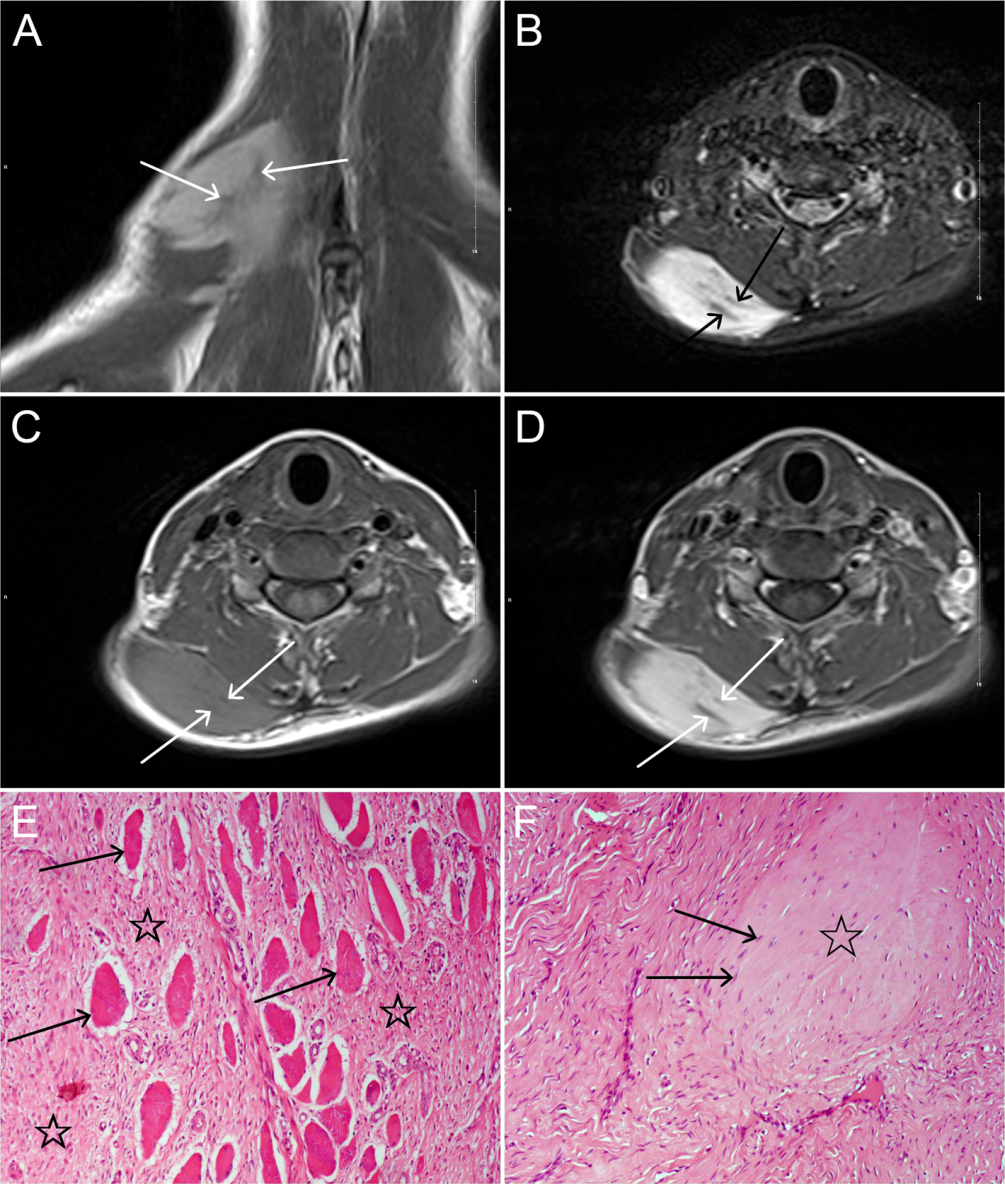
A young patient in her twenties with a myofibroma in the right trapezius. **a** and **b**: Coronal T2WI and axial FS T2WI demonstrate a lobulated, heterogeneous hyperintense mass with irregular strip hypointensities (arrows). **c**: On axial T1WI, the lesion appears as heterogeneous slight hyperintensity with irregular hypointense areas (arrows). **d**: On contrast-enhanced axial FS T1WI, the lesion shows inhomogeneous marked enhancement with non-enhanced hypointense areas. **e**: Photomicrograph (HE, × 100) shows a large number of interlacing bundles of spindle-shaped cells (☆) between normal muscles (arrows). **f**: Local hyalinization (☆) with a few degenerative tumor cells (arrows) can be seen (HE, × 100)

**Fig. 3:**
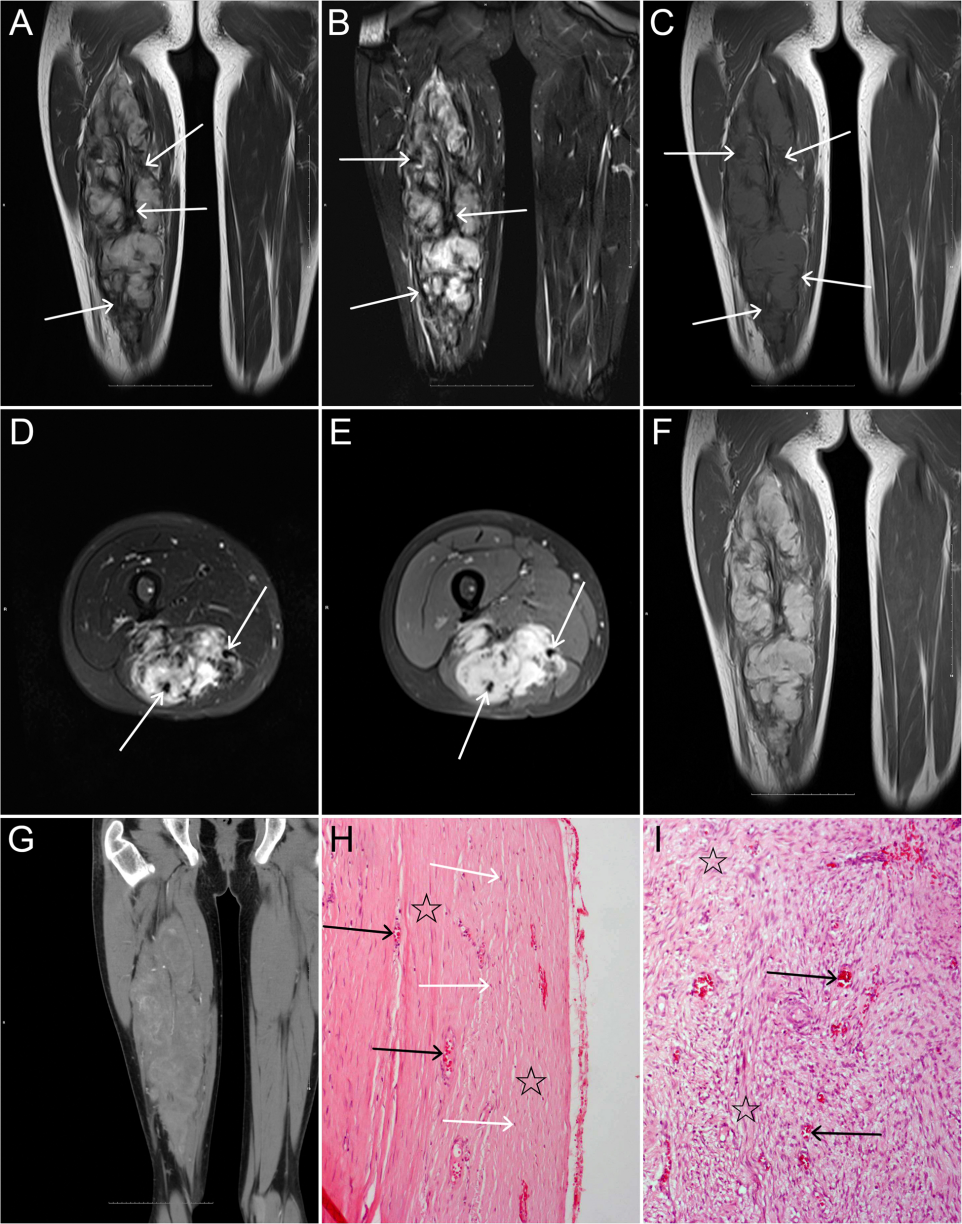
A young teenage patient with a myofibroma in the right thigh. **a** and **b**: Coronal T2WI and FS T2WI reveal a huge lobulated, heterogeneous hyperintense mass with irregular patchy, strip hypointensities, and incomplete pseudocapsule (arrows). **c**: Coronal T1WI shows heterogeneous slight hyperintensity with irregular hypointense areas (arrows). **d** and **e**: Axial T2WI and contrast-enhanced FS T1WI reveal the lesion encircling tendon (arrows). **e** and **f**: On contrast-enhanced axial FS T1WI and coronal T1WI, the lesion shows inhomogeneous marked enhancement with non-enhanced hypointense areas. **g**: On contrast-enhanced MDCT image, the lesion shows heterogeneous enhancement. **h**: Photomicrograph (HE, × 100) shows a pseudocapsule consisting of a large number of collagen fibers (☆) with a small number of spindle cells (white arrows) and vascular proliferation (black arrows) in the lesion. **i**: A large amount of vascular proliferation (arrows) and irregular fibrosis (☆) are present in the center of the lesion (HE, × 100)

The MRI features of 17 myofibromas in 15 patients are given in Table 2. In 17 tumors evaluated by MRI, 1 (5.8%) present as a round-like and well-defined nodule, all the other 16 (94.1%) tumors were lobulated masses with ill-defined margins. Their maximal diameter ranged from 1.5 to 28.5 cm (median 4.5, IQR 2.9–12.7). Among the 17 tumors, 4(23.5%) showed a combination of subcutaneous tissue and adjacent skeletal muscle invasion, and 2 of them showed bone destruction of phalanx on MRI and plain radiography; 1(5.9%) involved subcutaneous tissue and adjacent parotid gland; 11(33.3%) occurred in the skeletal muscle (Figs. 2 and 3) and 1(5.9%) located in the iliac fossa. Four (23.5%) out of the 17 tumors grew around the tendon (Fig. 3).

On T1WI, 14 (82.3%) tumors showed as heterogeneous slight hyperintensity, 1(5.9%) tumor showed homogeneous slight hyperintensity, and 2(11.8%) tumors showed heterogeneous hypointensity relative to the adjacent skeletal muscle. On T2WI, all 12(100%) tumors showed heterogeneous hyperintensity relative to the adjacent skeletal muscle, but relatively lower signal relative to the fat. On FS T2WI, all 17(100%) tumors showed heterogeneous hyperintensity. On T1WI, T2WI, and FS T2WI, there were 16(94.1%), 12(100%), and 17(100%) tumors with irregular strip or/and patchy hypointensities (Figs. 2 and 3) inside them, respectively, and pseudocapsule was found in 5(29.4%) tumors (Fig. 3). On FS contrast-enhanced T1WI, all 17(100%) tumors showed marked enhancement, but with irregular strip or/and patchy hypointensities within these tumors.

### Pathological and immunohistochemical findings and accuracy of radiological diagnosis

Histopathologic specimens were obtained from all patients, including 27 specimens from the final surgery and 2 from the biopsy. Histological features of these tumors were as follows: (1) interlacing bundles of spindle-shaped cells with a large number of collagen fibers were found in all 29 tumors, the tumor cells were large and presented tapered nuclei with elongated and eosinophilic cytoplasm; (2) spindle-shaped cells were polygonal or round to spindle in shape with hyperchromatic nuclei, scant eosinophilic cytoplasm in some tumor regions; (3) haemangiopericytoma-like vascular pattern could be identified in 20 (69.0%) tumors; (4) sometimes, necrosis, calcification, and hyalinization (Fig. 2) can be identified; (5) mitoses and nuclear pleomorphism were rare. (6) a pseudocapsule consisting of peripheral collagen fibers was found in 5 (17.2%) tumors (Fig. 3), which corresponds to the low signal pseudocapsule on MRI. The immunohistochemical findings as follows: 24 (82.8%) out of 29 tumors were positive for SMA, 2/29 (6.9%) for desmin, 1/29 (3.4%) for S^− 100^ protein, 6/29 (20.7%) for CD34, 29/29 (100%) for Calponin, and 4/29 (13.8%) for Ki-67. Preoperative imaging data of all patients were diagnosed as various benign, malignant tumors or inflammation, without a definitive qualitative diagnosis.

### Treatment outcomes and follow-up

Of the total 24 patients, 21 patients with 26 myofibromas were treated by complete excision and 1 of them (case 1) also received radiotherapy and chemotherapy, 1 patient underwent partial excision, and only tissue biopsy was performed in 2 patients. Twenty-one patients were followed up postoperatively for periods ranging from 6 months to 4 years (12.4 ± 6.8 months), 14 (58.3%) of them did not exhibit recurrence and no new lesion developed, but 7 (29.2%) were found to have local tumor recurrence and underwent reoperation. The remaining 3 patients were lost to follow-up. The age of the recurrent group (15.3 ± 7.0 years) was lower than that of the non-recurrent group (38.9 ± 14.3 years) (*P* = 0.001). There was no significant difference in the maximum diameter of the tumor between the recurrent group (median 5.6 cm, IQR 4.0–13.7) and the non-recurrent group (median 4.7 cm, IQR 2.6–13.5) (*P* = 0.572). The soft-tissue tumors without pseudocapsule (*n* = 11) on MRI were more likely to recur than those with pseudocapsule (*n* = 5) (*P* = 0.034).

## Discussion

In this report, we described the radiological findings of the largest series of myofibromas of soft tissue and skeletal lesions to date. Myofibromas usually exhibited heterogeneous density or signal intensity, with progressive moderate or marked enhancement. On MRI, most tumors had irregular strip or/and patchy hypointensities on all MRI sequences, and 5 of them showed a pseudocapsule at the periphery of the lesion and 4 tumors grew around the tendon.

Myofibromas could occur at any bone, the cranio-orbito-facial bones were most often involved with a solitary lesion, while, the other bone lesions were frequently observed with the multicentric type (17–77%) [2, 7, 15–18]. On plain radiography and CT, most skeletal lesions demonstrated well-defined, expansile, osteolytic, with or without a partial or poorly developed sclerotic rim which more commonly observed when the lesions start to heal [14, 18, 19]. Occasionally, periosteal reaction, cortical expansion, necrosis, and calcifications may be observed [15, 17]. Most intraosseous lesions of the mandible were unilocular radiolucency [20]. In our series, a mandible lesion simultaneously showed a partial sclerotic rim, periosteal reaction, and resorption of the external cortical bone, with marked enhancement. Vertebral body collapse was considered as another characteristic imaging findings of myofibromas [15, 21].

Nearly half of solitary myofibromas occurred in the deeper soft tissues of the aponeuroses, fascia, and skeletal muscle [2]. Soft-tissue lesions may be well-margin, or infiltrative and ill-defined on MRI or MDCT, and showed more invasive than bone lesions, and the erosion of adjacent bone could also be observed [12, 14]. In our study, we also found that 11 (40.7%) soft-tissue lesions simultaneously involved two or more tissues on MDCT or MRI, and 3 of them showed destruction of adjacent bone and 2 showed involvement parotid gland. Naffaa et al. [12] considered that the variable tumor margins could not predict microscopic invasion or cellular atypia. However, in our cohort, we found that a large number of tumor cells invade adjacent muscles at the edge of the soft-tissue lesions on pathology, which demonstrated lobulated mass with an infiltrative and ill-defined margin on MRI or MDCT. Our results indicated that the infiltrative tumor margins can predict microscopic invasion. Furthermore, we also found that the tumors without pseudocapsule were more likely to recur than those with pseudocapsule, and the age of the recurrent group was lower than that of the non-recurrent group. We suggested the absence of pseudocapsule and the younger age may be risk factors for tumor recurrence.

The density of the myofibromas of soft tissue was variable due to necrosis or calcification. Most soft-tissue lesions appeared with attenuation that is similar to or slightly higher than that of skeletal muscle on MDCT in our series and previous literature. However, some myofibromas presented with low density relative to the adjacent skeletal muscle on MDCT [12, 14, 19, 23], which was also observed in seven (7/15) of our cases. Necrosis or calcification could be seen in some cases. On MDCT, there were relatively few reports of the enhancement feature of soft-tissue myofibroma, most of them were ring-like or peripheral enhancement [12, 19, 23]. Naffaa et al. [12] described two soft-tissue lesions showed mild enhancement and all of them were slightly low or iso-density relative to the adjacent skeletal muscle on enhanced MDCT. In our study, we reported the largest number of cases of myofibromas with MDCT enhancement, and the enhancement value of the tumor was also quantitatively analyzed for the first time. Most of our cases presented heterogeneous moderate to the marked enhancement and progressive enhancement, and their actual enhancements were all higher than that of the adjacent skeletal muscle, and ring-like enhancement was rare. The reason for this difference may be that the vast majority of our cases were adults rather than infants and young children. Our study also did not observe a significant histological difference between lesions with moderate and marked enhancements.

As far as we know, the MRI signal characteristics of the myofibromas of soft tissue were also variable. Myofibromas were generally demonstrated as iso/hypointensity relative to the adjacent skeletal muscle on T1WI and hyperintensity on T2WI, and some cases showed a very high or low signal central region on T2WI due to necrosis, cystic, or mature myofibroblasts, or calcification [12, 14, 22–25]. However, unlike previous studies, in our series, most (82.3%) of lesions showed a slight hyperintensity on T1WI and 12 (100%) lesions demonstrated as a heterogeneous high signal but lower than fat on T2WI. There are several possible reasons to explain why some myofibromas can present slightly higher signal relative to the skeletal muscle on T1WI. First, the cell density of the myofibroma may be higher than that of the adjacent normal skeletal muscle. Second, the T1 relaxation value of the tumor cells may be lower than that of the skeletal muscle. Third, in our series, the calcification, necrosis, and cystic degeneration are rare, these can cause a decrease of the signal on T1WI, and a large amount of collagenization and fibrosis are also the important reasons for the low signal on MRI. In addition, we first described that 5 tumors showed a pseudocapsule at the periphery of the lesion and four tumors grew around the tendon. On enhanced MRI examination, the myofibromas may exhibit peripheral enhancement, nodular enhancement, homogeneous or heterogeneous enhancement, and mild to marked enhancement, and some cases showed no enhancement [12, 15, 16]. The peripheral/rim-like enhancement occurred mostly in infants and young children [14, 15, 22–24]. In our enhanced MRI studies, all 17 tumors appeared as a heterogeneous marked enhancement, without peripheral/rim-like enhancement. The signal characteristics of intraosseous lesions were similar to those of soft tissue lesions. However, there were also unusual imaging findings reported in the literature, Marret et al. [28] described a rare imaging finding of myofibroma that the lesion was disguised as humeral osteomyelitis on MRI.

Murphey et al. [14] and Salerno et al. [30] considered the different MRI signals of the myofibromas depending on the main pathological patterns of the tumors. Holzer et al. [15] suggested that the hypointensity on T2WI might be due to the high cellularity of the lesions. Naffaa et al. [12] considered that the extent of hyperintensity on T2WI indicated variable grades of cellularity, collagenization, and myxoid changes, and suggested that the pattern of enhancement on MRI does not correlate to any specific histopathology grade. Our pathological examinations revealed that tumor cells were interlaced with collagen fibers and fibrosis in all tumors. Correspondingly, on T2WI and FS T2WI, we found that the hyperintense area was interspersed with irregular strip or/and patchy hypointensities in all lesions. Interestingly, 5 of them showed a pseudocapsule consisting of peripheral collagen fibers on pathology which was exactly corresponding to the pseudocapsule at the tumor periphery on all MRI sequences. Therefore, our results demonstrated that the hypointense areas on all MRI sequences may be indicative of interlacing collagen fibers and fibrosis rather than calcification, in other words, the interlacing collagen fibers and fibrosis should be the dominant factors for these hypointensities.

This study had several limitations. First, this is a retrospective study, which means that there were no perfect imaging protocols, different MDCT and MRI equipments and techniques were used. Second, the relatively smaller sample size is another limitation. Third, owing to a lack of detailed clinical information in this group of cases, all cases were examined only by local imaging examinations but not by whole-body examinations, so the number of multifocal and generalized lesions may be underestimated. However, these problems are unavoidable due to the rarity of this type of tumor, and this should not have a significant impact on the radiological characteristics studied.

## Conclusion

In conclusion, myofibromas are usually characterized by heterogeneous density or signal intensity, with moderate or marked enhancement. Our study is the first to describe that myofibroma could present a pseudocapsule at the periphery of the lesion and grow around the tendon, and the irregular strip or/and patchy hypointensities could be noted on all MRI sequences. Although these imaging characteristics of myofibromas are still nonspecific and the final diagnosis must be made by pathology, we believe that the results of this report may be helpful in the diagnosis of tumors and in differentiating them from other bone and soft tissue tumors on imaging.

## Data Availability

The datasets used and/or analyzed during the current study are available from the corresponding author on reasonable request.

## Abbreviations

MDCT: Multidetector computed tomography
MRI: Magnetic resonance imaging
T1WI: T1-weighted imaging
T2WI: T2-weighted imaging
FS: fat-suppressed
HU: Hounsfield units
IQR: Interquartile range
TR: Repetition time
TE: Echo time
